# Effect of A New Housing System on Skin Lesions, Performance and Soiling of Fattening Rabbits: A German Case Study

**DOI:** 10.3390/ani9090650

**Published:** 2019-09-04

**Authors:** Sally L. Rauterberg, Joana Bill, Sarah Kimm, Nicole Kemper, Michaela Fels

**Affiliations:** Institute for Animal Hygiene, Animal Welfare and Farm Animal Behavior, University of Veterinary Medicine Hannover, Foundation, Germany, Bischofsholer Damm 15, Hannover D-30173, Germany (J.B.) (S.K.) (N.K.) (M.F.)

**Keywords:** growing rabbits, welfare, floor type, enrichment, stocking density, group size

## Abstract

**Simple Summary:**

The present study evaluated a new housing system for fattening rabbits which, on the one hand, complied with new German legal requirements and on the other hand was expected to provide some benefits in terms of animal welfare compared to previously established cage systems in Germany. Fattening rabbits kept in the new housing system showed a lower incidence of injuries and higher daily weight gain than rabbits kept in conventional cages on the same farm, which may indicate increased welfare in this regard. However, an increased mortality rate and hygienic challenges posed by the new system may indicate impaired welfare and are not acceptable in this form. Finally, both the conventional and the new system were deficient in terms of animal welfare. Further research is necessary to find a housing system that complies with German legislation and keeps rabbits in good health and welfare simultaneously.

**Abstract:**

The aim of the present study was to develop and evaluate a new housing system for fattening rabbits. Data were collected on a farm with rabbits housed either under new conditions (NC) or established (conventional) conditions (CC). NC housing was characterized by large groups (Ø 58 rabbits, max. 12 rabbits/m^2^), slatted plastic floor (11 mm slats and 11 mm gaps), elevated platforms with partly solid floor, boxes and different enrichment materials. CC rabbits were kept in small groups (eight rabbits, 23 rabbits/m^2^) in cages with wire-mesh floor, an elevated platform, a box and one gnawing stick. Skin lesions and weight gain of 524 rabbits, cleanliness of their hind feet as well as their mortality and morbidity were investigated from weaning to slaughter in five batches. The evaluations showed higher daily weight gain (46.3 ± 6.0 g vs. 43.1 ± 5.5 g) and final weight (2878 ± 328 g vs. 2707 ± 299 g), as well as a lower cumulative lesion score at the middle of the fattening period in NC than in CC rabbits. Nevertheless, cleanliness of hind feet was assessed to be worse and mortality was higher in the NC housing. The NC system provided some benefits in terms of animal welfare compared to the conventional system, but hygienic challenges posed by this system make further adjustment necessary.

## 1. Introduction

Increased demands on animal welfare require adaptations of existing housing conditions on commercial rabbit farms. This has increasingly led to political efforts to adjust the legal requirements for rabbit husbandry in Germany and other countries [1,2]. On commercial farms, fattening rabbits are usually reared in pairs or small groups in cages with wire mesh floor and a roof. There, stocking densities range from 14 up to 22 rabbits/m^2^ [3,4]. The barren environment and spatial restriction in such cages are often associated with technopathies [5], increased abnormal and stereotypic behavior or aggression [3]. This may indicate reduced welfare and led to various approaches aimed at improving housing conditions according to rabbits’ needs. For instance, fattening rabbits reared in large groups in pens instead of small groups in cages are less restricted in their movements [6,7]. However, even if the time spent moving and exploring and social behavior increases in larger groups [8,9,10,11], some authors also found an impaired growth performance [6,7,8,12,13,14,15,16,17] as well as an increase in aggression and injuries in larger groups [9,10,18,19,20,21]. In contrast, other authors did not find any effect of the group size on growth performance or injuries [22], or even fewer injuries [6] and a higher daily weight gain in larger groups [23]. The mortality was mostly unaffected by group size [7,15,16,18], but sometimes increased in larger groups [8,14]. Nonetheless, not only the group size but also the stocking density seemed to play a role, as especially larger groups along with a higher stocking density led to an impaired growth performance [2,17,24]. If the space per animal was increased while group size remained the same to obtain a lower stocking density, an improved daily weight gain and higher final weight were observed [25]. However, here again research is inconclusive, as other studies found no effects on growth performance [26,27] or mortality [28].

Furthermore, the commonly used wire mesh floor is suspected to have a negative impact on animal welfare [5,29,30] and animals which were able to choose were observed to prefer a plastic net floor [10,31,32,33]. In contrast, the preference for deep litter in fattening rabbits was low [34,35] and straw-bedded pens were often associated with lower feed intake and weight gain, as well as a higher mortality [8,24,34]. On the other hand, for different versions of plastic net floors, no negative impact on performance [15,27,28,36], mortality [15,28], injuries [36] or behavior and reactivity of the rabbits [11,27] was detected in comparison to wire net floors. Even if the shape and degree of perforation may have an influence on cleanliness and hygienic aspects of plastic net floors [1,37,38], it seems to be promising for further usage in terms of animal welfare, especially for the reason that fattening rabbits prefer this kind of floor and it did not show any disadvantages concerning any productive traits [15].

A possibility to enrich the rabbits’ environment is to provide them with different materials, such as gnawing sticks. These were observed to reduce aggressive behavior [11,15], injuries [10,11,39] and abnormal behaviors such as bar gnawing [40,41,42,43]. Some authors also found a positive impact of wooden sticks on daily weight gain and slaughter weight [40], and temporarily higher daily weight gain in enriched pens with a platform, hiding box and gnawing material [44].

These findings indicate that current conventional cage systems may impair animal welfare, which is not acceptable from both an animal scientist’s and a societal point of view. However, the lack of appropriate, practicable and law-compliant rabbit housing systems for commercial farms in different countries makes progress difficult regarding this aspect [2]. Thus, in the present study, a novel housing system was developed and implemented on farm. In particular, this housing should comply with new legal requirements in Germany. By providing increased space per animal, slatted plastic floor, environmental enrichment and structural elements it was also expected to have a positive impact on the welfare and performance of fattening rabbits. On the other hand, this system may bring some challenges from a hygienic point of view by providing a floor with a low degree of perforation and pens made to keep large groups. Whether the new housing system is indeed beneficial for rabbits’ welfare or not was evaluated by collecting and analyzing data on performance, health and hygienic aspects in rabbits kept in the novel housing system and rabbits kept in the established conventional system on the same farm.

## 2. Materials and Methods

### 2.1. Animals and Housing

The experimental setup was approved by the Animal Welfare Office of the University of Veterinary Medicine Hannover, Foundation, Germany, with protocol TVO2017B4. 

It was carried out on a German commercial rabbit farm keeping about 600 rabbit does (Hyplus PS 19, Hypharm S.A.S., France) and their offspring (Hyplus PS 19 × PS 59, Hypharm S.A.S., France). For this study, fattening rabbits were kept in five batches either under housing conditions which were widely used and established in Germany (conventional conditions = CC) or under new housing conditions (NC), which were in accordance with new German animal welfare regulations.

**Figure 1:**
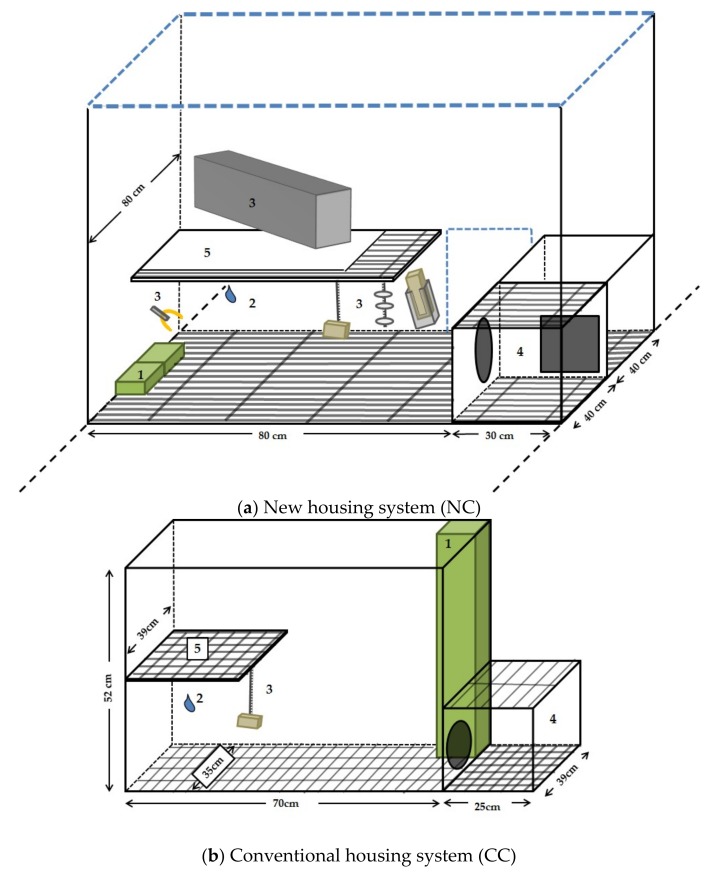
Unit of the new (NC) (**a**) and the conventional housing system (CC) (**b**) with feeder (1), nipple drinker (2), environmental enrichment (3), box (4) and elevated platform (5). After weaning, a NC pen compromised six NC units in a row. The blue dashed lines indicate open walls.

NC rabbits were born in the system, which consisted of 24 single units for does and their litters (Figure 1). In these single units, kits were reared in litters and weaned at an age of 31 days. At weaning, the does were removed and up to six litters were mixed by opening doors between six adjacent units, resulting in an NC pen for fattening. Thus, large groups with a maximum of 65 rabbits (58 ± 7 rabbits, max. 12 rabbits/m^2^) were formed. Each single unit measured 80 cm (length) × 80 cm (width) with an open top and slatted plastic floor (11 mm slats and 11 mm gaps). Additionally, there was a box with a flat roof, made of the same slatted plastic floor (30 cm × 40 cm × 27 cm (height)). The roof could be used as an elevated platform by the rabbits while the box served as a place to retreat. Next to the box, a small additional floor space (30 cm × 40 cm without roof) was available. Each unit also provided an elevated platform with partly solid floor (60 cm × 55 cm × 37 cm) and environmental enrichment in the form of a plastic tube and gnawing materials such as a piece of wood attached to a chain, a wood in a holder, a chain with plastic elements and a cotton rope. The walls of the NC units were made of wire-mesh (Figure 1).

CC rabbits, on the other hand, were born in conventional wire cages (70 cm × 50 cm × 30 cm) with a nestbox (30 cm × 28 cm × 28 cm). After weaning at the age of 31 days, they were moved to other cages into groups of eight mixed from two litters each (23 rabbits/m^2^). These groups were kept in 22 cages measuring 70 cm × 35 cm × 52 cm with closed walls. Each cage had a wire mesh floor (12 × 96-mm holes and 3-mm wire diameter) and was equipped with an elevated platform (32 cm × 39 cm × 27 cm) and a box (25 cm × 39 cm × 24 cm, wire mesh roofing), both with slatted plastic floor (16 × 16 mm holes and 7 mm slats). Additionally, one piece of wood attached to a chain was provided in each cage as the gnawing material (Figure 1).

Rabbit does on the farm were vaccinated against RHD (Rabbit Haemorrhagic Disease), enteritis and pasteurella multocida. Fattening rabbits which were investigated in the present study were not vaccinated. Fattening rabbits remained in the systems until slaughter at the age of 78 days. In both housing systems, a commercial pelleted diet, chopped hay and water from one nipple drinker per cage and two per pen were available ad libitum. Both systems were equipped with a negative-pressure ventilation system and during the daytime with artificial lighting. In the CC group, this lasted from 7:00 to 19:00 and in NC, from 6:00 to 19:00 with dawn from 6:00 to 6:30 and dusk from 18:30 to 19:00. Manure removal in NC housing was carried out daily by means of a manure belt placed below the pens. In CC housing, manure was stored in concrete pits below the cages which were emptied after each batch.

### 2.2. Data Collection

At days 31, 35, 52 and 77 of life, following data of 284 fattening rabbits kept in NC housing and 240 kept in CC housing were collected at the individual level:

1. Sex was determined and each rabbit was marked individually with animal paint spray.
2. Presence and severity of skin lesions were determined by scoring different parts of the body (ears, head, body, tail, limbs, genitals and belly) using the scoring system shown in Table 1. Subsequently, a cumulative lesion score for each animal was calculated as the sum of the different body regions (Minimum: 0, Maximum: 28).
3. Body weight was recorded via a PCE-PB 60N scale and based on that, daily weight gain was calculated.
4. Rabbits’ hind feet were checked for the development of pododermatitis and scored for soiling (Table 1).
5. Rabbits suffering from rhinitis, conjunctivitis or diarrhea were recorded after clinical examination.
6. Dead animals were recorded and the mortality rate was calculated. No further investigations on the causes of death were made.

### 2.3. Statistical Analysis

The assessed data were analyzed using R 3.6.0 [45]. The level of significance was set at p < 0.05. All examined data were initially assessed for normal distribution using histograms and Shapiro-Wilk tests. Body weight and weight gain were analyzed using linear models which included the fixed effects housing, sex and batch. Logistic regressions were performed to analyze differences in the number of diseased and injured rabbits with housing, sex and batch as fixed effects. In addition, mortality was analyzed using logistic regressions with housing, sex, batch and the recorded diseases as fixed effects. Odd’s ratios (OR) were calculated using Fisher’s exact test. The cumulative lesion score was tested with a generalized linear model with housing, sex, observation day and batch as fixed effects. Soiling of hind feet was analyzed using an ordered logistic regression with housing, observation day and batch as fixed effects using the R packages MASS (Support Functions and Datasets for Venables and Ripley’s MASS) [46], lmtest (Testing Linear Regression Models) [47] and AER (Applied Econometrics with R) [48].

## 3. Results

### 3.1. Skin Lesions and Pododermatitis

**Figure 2:**
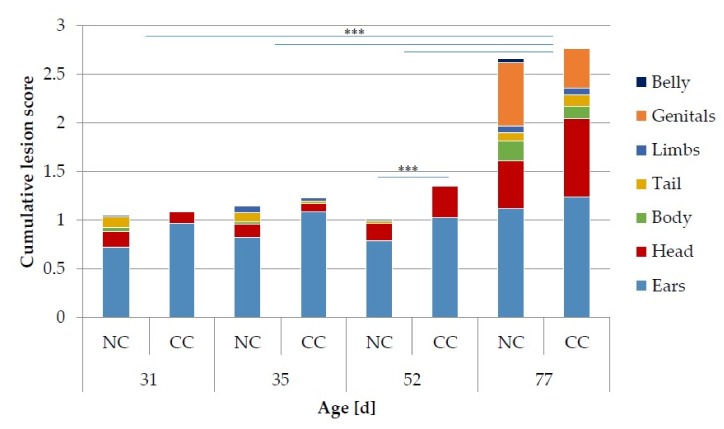
Cumulative lesion score and mean lesion score for the different body parts of rabbits kept under conventional (CC) and new conditions (NC) at different observation times, *** *p* < 0.001.

Overall, a mean cumulative lesion score of 1.5 (±1.6 (SD), median: 1, minimum: 0, maximum: 12) in all groups for all observation times was observed. A total cumulative lesion score of zero during the entire fattening period was more often assessed in NC than in CC rabbits (OR = 5, *p* < 0.01). During the entire fattening period, effects of housing (*p* < 0.01), sex (*p* < 0.001), observation day (*p* < 0.001) and batch (*p* < 0.001) on the cumulative lesion score were observed. At each observation time, the lesion score was lower in rabbits from NC than from CC. However, a significant difference was only found at day 52 of life (NC = 1.0 ± 1 (SD), median: 1, minimum: 0, maximum: 5 vs. CC = 1.4 ± 0.9 (SD), median: 1, minimum: 0, maximum: 4, *p* < 0.001). At the end of the fattening period, a significant increase in the cumulative lesion score was noted in both housing systems with a higher score at day 77 (2.7 ± 2.2 (SD), median: 2, minimum: 0, maximum: 12) than at days 31, 35 and 52 of life (*p* < 0.001, Figure 2). Additionally, male rabbits showed a higher lesion score than females at day 77 (3.2 ± 2.4 (SD), median: 3, minimum: 0, maximum: 12 vs. 2.3 ± 1.9 (SD), median: 2, minimum: 0, maximum: 10, *p* < 0.001).

**Figure 3:**
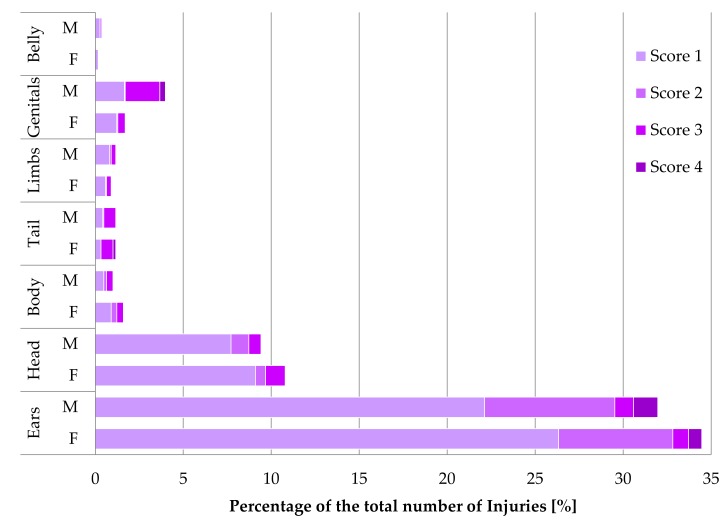
Distribution of the total number of skin lesions (% N = 1880) at different body parts considering the given scores 1–4 (Table 1) in female (F) and male (M) rabbits from both housing systems.

Considering all injuries that occurred during the entire fattening period (N = 1880), most of them affected the ears (66%) and were mostly assessed with score 1 (73%, Figure 3). The second most frequent injuries occurred at the head (20%), also mainly assessed with score 1 (83%). Both animals with injuries to the ears and head were more frequent in CC than in NC housing (*p* < 0.001). Injuries to the body, tail, limbs and belly were hardly observed (3%, 2%, 2%, 1%, respectively). Neither an effect of housing nor of sex on the number of injured rabbits at these body parts were detected (*p* > 0.05), but an effect of batch in terms of body, tail and belly (*p* < 0.05). Lesions at the genitals (6%) increased at the end of the fattening period with male rabbits being more often affected than female rabbits (OR = 3, *p* < 0.001). Lesions at the genitals assessed with a higher score than 1 were initially observed at day 77. Additionally, in males, these injuries were more often assessed with score 3 (49%) and 4 (8%), while score 4 was never observed in females (Figure 3). Pododermatitis was not observed until slaughter in both housing systems. Only a small hairless area was found at the hind feet of all rabbits in both housing systems.

### 3.2. Body Weight and Daily Weight Gain

Body weight and daily weight gain of rabbits from NC and CC housing are shown in Table 2 depending on the observation day or period. Daily weight gain from day 31 until day 77 was affected by housing (*p* < 0.001), sex (*p* < 0.05) and batch (*p* < 0.01). NC rabbits showed higher daily weight gain from day 31 until day 77 of life (*p* < 0.001) and higher final weight on day 77 of life (*p* < 0.001). Especially in the second half of the fattening period, an effect of housing system (*p* < 0.001) and sex on the daily weight gain was observed (*p* < 0.01). In contrast, in the first half, neither housing nor sex had an effect (*p* > 0.05), but only batch (*p* < 0.001). When considering all rabbits from both housing systems, female rabbits showed higher daily weight gain than male rabbits throughout the entire fattening period (45.2 ± 6 g vs. 43.8 ± 6 g, *p* < 0.05) and especially in the last half of the fattening period (46.2 ± 8 g vs. 43.4 ± 9 g, *p* < 0.01).

### 3.3. Soiling of Rabbits’ Hind Feet

**Figure 4:**
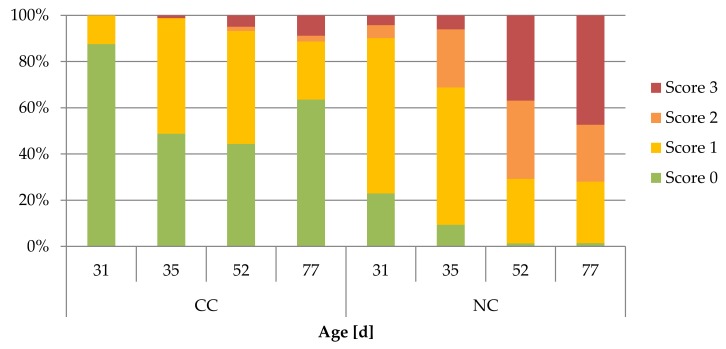
Distribution of rabbits’ hind feet soiling assessed with scores 0–3 (Table 1) under conventional (CC) and new housing conditions (NC).

The degree of soiling of rabbits’ hind feet was influenced by the housing system (*p* < 0.001), the observation day (*p* < 0.001) and batch (*p* < 0.001). There, CC rabbits were assessed as being cleaner than NC rabbits at any observation day (*p* < 0.001). In NC housing, hind feet scored as clean (score 0) were found less frequently (23%) at the weaning day (31d), while score 3 (4%) was already observed at this day. On the contrary, the feet of CC rabbits only showed score 0 (88%) and score 1 (12%) at day 31. In both housing systems, a significant increase in soiling was detected already four days after weaning at day 35 (*p* < 0.001) and again until day 52 (*p* < 0.05). At the end of the fattening period at day 77 of life, only 1% of NC rabbits received score 0, but 47% score 3. In CC rabbits, 64% were assessed with score 0 and 9% with score 3 at day 77 (Figure 4).

### 3.4. Morbidity and Mortality

Morbidity and mortality in both housing systems are shown in Table 2. The total morbidity (number of animals suffering from at least one of the recorded diseases) was not associated with housing conditions, sex or batch (*p* > 0.05), but housing tended to influence the occurrence of rhinitis (*p* = 0.051). No differences in the ratio of conjunctivitis and diarrhea were found (*p* > 0.05). The total mortality was higher in NC than in CC housing (OR = 2, *p* < 0.001), also with a significant effect of batch (*p* < 0.001), but no effect of sex (*p* > 0.05). Especially in the first half of the fattening period, an effect of housing (OR = 2.5, *p* < 0.01) and batch (*p* < 0.001) on mortality was observed, while in the second half, only batch still had a significant effect (*p* < 0.01). While diarrhea and rhinitis had a significant effect (*p* < 0.05) on the mortality, no effect of conjunctivitis (*p* > 0.05) was observed.

## 4. Discussion

Considering conventional cage systems, the development of new housing systems for fattening rabbits seems to be necessary in terms of animal welfare. As a reaction, different countries also specified their animal welfare laws, with requirements for the housing conditions of rabbits [1,2]. Therefore, the present new housing system should comply with current legal requirements in Germany [49] which have had to be implemented on commercial farms since 2019. Based on various characteristics, the new housing system was expected to have a positive impact on animal welfare. An increased space allowance as well as structural and enrichment elements may provide an advantage over established conventional cages, especially from a behavioral point of view [6,8,9,10,11,15,40,41,42]. This has already been confirmed in preliminary results about rabbits’ behavior in the present housing system [50]. Higher daily weight gain and fewer injuries shown in the results of the present study verified a positive impact of the newly developed system. Nevertheless, when judging its suitability, a wide range of aspects has to be considered. Thus, data on hygienic conditions and animal health as well as performance data of rabbits were collected. However, results showed that the new housing system did not sufficiently fulfill all claims on animal welfare.

### 4.1. Skin Lesions and Pododermatitis

In contrast to other investigations [9,10,18,19,20,21], injuries occurred less frequently in NC housing with larger groups than in CC housing with small groups. One reason for this may be the difference in the available space per animal. A lower stocking density seems to be beneficial in terms of the occurrence of injuries [2]. Due to the lack of space in the CC housing and the high stocking density, the rabbits there may more often trample on each other accidentally. To solve this problem it was recommended to reduce the stocking density and to provide suitable resting areas [6]. Moreover, in terms of aggression, it would be beneficial to offer possibilities to avoid the contact to pen mates and to withdraw if necessary [51]. In this context, the new housing system offers some advantages because it provides more places to hide and more space for the animals to stay away from each other. The increased supply of enrichment may also have a positive impact on the amount of injuries, as enrichment was observed to reduce aggression and injuries in previous studies [10,11,15,39,43].

Looking at the injuries in more detail, it is interesting to note that most injuries occurred to the ears, which confirms the findings of previous studies [22]. This could be due to the lack of fur there, which, on the one hand, may protect the skin against abrasions. On the other hand, this makes it easier for the observer to clearly detect even the smallest lesions. Different authors have investigated the occurrence of ear lesions as an indicator of wellbeing and stress. Most of them used it as sign of occurring aggression in the group [18,20,52] and thus as a sign of stress in the groups containing aggressive individuals [18]. Others explained it as an effect of the housing conditions [6]. In the present investigations, we assumed that this kind of lesions may have its origin both due to housing and social interactions. Most of the present ear lesions were minor (score 1) and may have occurred due to animals trampling on each other or due to the housing environment itself. Aggression cannot be excluded as a cause, even if this is more likely in the case of more severe injuries and wounds (scores 2 and 3). This also applies to the partial or total loss of ears (score 4), but this was rare and is assumed to mostly have its origin in the earlier rearing period. However, a high incidence of ear lesions with wounds and partial losses occurred in one batch at the end of the fattening period, with a very likely origin in ear biting between pen mates in the new housing system. Apart from that, it should be assessed positively that rabbits with ear injuries were less common in NC compared to CC rabbits, which could indicate a lower stress level within these groups. The same applies for lesions at the head, which occurred less frequently at the ears but were also less common in the new system. All other body parts were seldom affected and thus, no difference between the housing systems was observed. In both systems, an increase in the cumulative lesion score was detected at the end of the fattening period, which agrees with other investigations [18,22,25,51,52,53]. This may be due to some individuals reaching sexual maturity, which can lead to an increase in aggressive behavior between the animals [10,18,53]. Additionally, at the end of the fattening period, more injuries were observed in male rabbits compared to female rabbits, since male rabbits often show more aggression and injuries at the end of the fattening period [2,21,52,53]. This assumption is also supported by the increase in injuries to the genital area, similarly to other investigations [22]. These occurred especially in male rabbits [21,54] and also with a higher severity. Injuries to the genital area occurred more often in the new compared to the conventional housing system, even if this difference was not significant, probably due to the small total number of genital injuries. One reason may be that there were single individuals who had already reached sexual maturity and were more aggressive. These single rabbits could consequently injure more rabbits in larger groups than in smaller groups [10,18]. Additionally, with more rabbits, the probability of more animals reaching sexual maturity increases. Similarly, previous investigations showed more injuries to the genitals in large groups than in small groups [55]. The problem of a higher incidence of injuries can be avoided by slaughtering fattening rabbits before sexual maturity, so that dominance and sexual behavior leading to aggression do not occur [7,56]. In contrast to the results based on the end of the fattening period, weaning and mixing different litters did not seem to have an effect on the frequency of injuries. Thus, no increase in the lesion score was observed in the first four days after weaning, i.e., from day 31 to 35. 

In this study, the development of pododermatitis was detected neither in rabbits reared on wire mesh nor on plastic net floors during the entire fattening period. This result was expected due to the early slaughter age of fattening rabbits [4]. Nevertheless, in all investigated rabbits, small furless areas already existed at the hind feet. This leads to the assumption that in case of a longer housing period, pododermatitis will develop in both housing systems. As there seems to be a correlation between pollution at the hind feet and the occurrence of pododermatitis [38,57], a fast and severe development is assumed in the new system. However, as wire mesh flooring is known to increase the occurrence of footpad lesions as well [58], a development of severe pododermatitis is also expected in CC housing in case of a longer housing period. Even if CC housing also provides partly slatted plastic floor, which can reduce the severity of pododermatitis [59], the high stocking density in CC does not allow all rabbits to adequately use these alternative floor areas.

### 4.2. Body Weight and Daily Weight Gain

Rabbits from NC housing reached higher daily weight gains and final body weights than CC rabbits. On the contrary, different studies showed that more space and locomotion possibilities usually had a negative impact on performance due to greater physical activity [8,15,60]. Additionally, most previous studies showed an impaired growth performance in larger groups [6,8,12,13,14,15,16,17,55]. However, different factors in the new housing system may have a positive effect on the performance. First, nearly all animals were allowed to feed at the same time due to a close animal/feeder space ratio. Thus, at some observation times, up to 15 rabbits were observed at one of the six feeders in the new housing system, while only one or, rarely, two rabbits were observed at the feeder in the conventional cages at the same time. This may have enhanced the feed intake and therefore, the body weight gain. In general, the lower stocking density may also have had a positive effect on the performance since some studies also showed a better weight gain in rabbits kept at a lower density [24,25]. Especially in the second half of the fattening period, CC rabbits showed an impaired growth performance. Since they were older and heavier, taking up more space for themselves, the spatial restriction in CC housing may have created less comfortable conditions, resulting in an impaired growth performance [24]. Additionally, it was observed that higher stocking densities led to decreased feed intake in the last weeks of fattening, probably due to the reduced space available for movement [26,56]. 

The more enriched environment in NC housing also may have improved the performance, as enrichment was already observed to have a positive impact on the daily weight gain and slaughter weight of fattening rabbits [40,44]. There, the enrichment may have had a stress-reducing effect and therefore, may have improved their biological functioning, leading to an increased growth rate [40].

An expected, different stress level during weaning may also have had an effect on later development during the growth period. While CC rabbits in the present study were separated from the doe and moved from the rearing cages to the fattening cages at the same time, rabbits from the new system did not have to leave their rearing housing, i.e., their familiar environment. This may have led to an increased stress level in caged rabbits and therefore, to an impaired growth performance after weaning. Nevertheless, the lack of a difference in daily weight gain directly after weaning makes this theory rather unlikely. An effect of the floor type was not assumed, as different floors did not show any effect on daily weight gain or final weight in previous studies [15,27,36].

Furthermore, the positive effect of housing on daily weight gain has to be discussed in relation to the high mortality rate. Only the surviving animals were weighed at the end of the fattening period. Assuming that the daily weight gain was correlated with the mortality rate and animals with less daily weight gain had a higher risk of dying, we have to assume that especially the heavier animals survived until the end of the fattening period. Even if this problem was the same in the conventional housing, the mortality rate was lower there. Supporting this hypothesis, a significant difference in daily weight gain was only observed in the second half of the fattening period, not in the first. However, the fact that the highest mortality rate occurred in the first half of the fattening period weakens this assumption.

### 4.3. Soiling

Even if slatted plastic floor was often preferred by the rabbits in previous studies [10,31,32,33] and also assessed as being more comfortable [30], the plastic net floor used in the present study brought some disadvantages. It is assumed that its perforation and surface structure led to strong soiling, which was reflected in the soiling of the animals, since this is directly related to a dirty floor [1,61]. Depending on the perforation degree and the shape of the floor, other authors also found an increased soiling in fattening rabbits on perforated plastic floors, which is more severe on less perforated floor [1,38,62]. In the present study, especially the elevated platform had to be taken into account. The attached solid floor may foster dirt accumulation [63,64]. Other authors also found a higher degree of pollution with a similar degree of perforation of the elevated platform [61]. Besides the hygienic disadvantages, there are also investigations showing that the preference of rabbits in choice tests decreases for floors which were more soiled, even if it was a less perforated slatted plastic floor [57].

Even if most authors did not show a negative impact of slatted plastic floor on the mortality [15,28,65], a relation between mortality and the soiled slatted plastic floor is most probable considering the present results. Direct contact with excreta may be similar to the problem in rabbits raised in straw-bedded pens [8], which is often related to infections and a higher mortality rate [8,24,34]. The present floor had 11 mm slats and 11 mm gaps and the elevated platform had a degree of perforation of only 15% to comply with German law [49]. This has to be considered when comparing the present results with other studies, since the degree of perforation was lower than in some of the previously studied floors [15,28]. Nevertheless, an association between a faster pollution of plastic net floor and more health problems and an increased mortality has already been suggested [15]. Additionally, it is assumed that the lower the perforation degree, the higher the dirtiness of floor, and consequently, the animals, resulting in a higher mortality rate [1].

However, most studies did not assess the cleanliness of floors or animals systematically, even if it may be an important factor influencing animal health and welfare. As such, this would be useful as an indicator of animal welfare and important for the evaluation of new floors [1].

Nevertheless, even if wire net is rated as the most hygienic option [37], it is not an appropriate alternative due to other welfare issues [5,29,30,58]. Therefore, further improvement and the development of a suitable plastic floor are necessary. Additionally, a regular intermediate cleaning could be recommended. Preliminary results of our own further investigations showed that a different kind of plastic floor with the same slat and gap width but with a slightly rounded surface can reduce the degree of soiling [66]. Thus, a rounded surface was already observed to improve cleanliness since urine drained better and the floor dried more quickly [38]. Additionally, modifying the platform by rearranging the perforation while maintaining the 15% perforation led to an improvement in the hygienic conditions [66]. Nevertheless, a more perforated platform would probably be beneficial [61]. On the other hand, a higher perforation of the elevated platform may lead to the problem that droppings and urine may fall onto other animals or into feeders if there is no manure tray underneath [63,64].

### 4.4. Morbidity and Mortality

Morbidity and mortality may not only be influenced by hygienic conditions but also by the different group sizes. As previously described, a higher mortality may be associated with larger groups due to an increased infection pressure [8,14]. Even though mortality rates in rabbits may occur in the range of the present ones [4], the mortality was unacceptably high in both housing systems from a welfare as well as from a productive point of view. Thus, a general health problem was assumed on the entire farm during the time of investigations. At this point, it had to be taken into account that the present investigations were made on one single farm. Investigating the same housing system on another farm may have had an effect on the resulting evaluation. Especially with regard to the health status, as there were health problems detected in both housing systems on the investigated farm. However, the large groups and the lack of hygiene in the new system may have reinforced this already existing problem. A solution for this problem is necessary and seems to be possible, as other investigations neither found a similar high mortality nor an increased one in larger groups or housings with perforated plastic floor [7,8,14,15,28,65]. After all, there are also investigations about keeping small or even large groups on flooring with a perforation degree similarly to that used in the present study (10-mm slats and 10-mm gaps). These did not show any negative impacts and were assessed as being suitable in practice [1,65]. However, these rabbits were housed at a lower stocking density and without an elevated platform with similar low perforation [1,65]. 

The common but inconsistent practice of preventive medical treatments may also have an influence on morbidity and mortality and impedes the comparability of different studies [15,28,37]. All these factors must be taken into account when evaluating mortality in rabbits and its causes to subsequently improve these factors and reach an adequate health status.

However, none of the obvious diseases were significantly more frequent in NC than in CC housing. These results are similar to other studies, which showed no significant differences in the occurrence of diseases but in the mortality rate. A possible explanation may be the fact that animals in a cleaner environment are more likely to recover from a disease than animals in a dirty environment [1]. Rhinitis may be an exception, as it tended to occur more often in NC than in CC. Since there was no pronounced difference in barn climate (temperature, humidity, air flow) between NC and CC housing, the cause may be the increased infection pressure in NC due to the large groups and the soiled environment. Unlike diarrhea, rhinitis may less frequently cause a fast death of rabbits and thus, a higher prevalence of rhinitis could be detected at the four observation times in NC. Conjunctivitis, on the other hand, occurred not often enough to find any difference.

## 5. Conclusions

A general assessment of a housing system for rabbits concerning its animal friendliness is quite complicated, especially due to sometimes contradictory results when evaluating different housing systems with various factors of influence. The aim of the present study was to evaluate a newly developed housing system as a whole which corresponded to new German legislation. This did not allow a dissociation of the respective effects of group size, floor type, stocking density, group composition or environmental enrichment.

Overall, the new developed system may provide some benefits in terms of animal welfare compared to the established conventional housing system. Nevertheless, the hygienic challenges posed by this system make further adjustment necessary. An improvement of the floor design may avoid excessive soiling of pen structures and animals and thus, may lead to better results concerning the general health status. Finally, neither the newly developed nor the conventional housing system sufficiently fulfills rabbits’ needs, for various reasons.

As there is no commercially available housing system meeting all new legal requirements in Germany, further research is urgently needed. This should assess rearing methods which enable farmers to keep their animals in accordance with existing legislation while considering the complex equilibrium between growth, behavior and an adequate health status. In particular, studies should also focus on investigations on different farms to draw sufficient and meaningful conclusions about animal welfare.

## Figures and Tables

**Table 1:** Scoring systems to evaluate the severity of skin lesions and soiling of rabbits’ hind feet.

Score	Skin Lesions	Soiling
0	Skin intact	Clean and dry
1	Small skin lesions (≤ 5 < 1 cm)	Dry soiled ≤ 50% of total feed area
2	Severe skin lesions (≤ 5 > 1 cm, or > 5 < 1 cm)	Dry soiled > 50% of total feed area
3	Wounds or > 5 > 1 cm	Wet soiled
4	Partial or total loss of tissue	

**Table 2:** Body weight, daily weight gain, mortality and morbidity of fattening rabbits from new and conventional housing conditions.

Age (d)	NC ^1^	N ^2^ (NC)	CC ^3^	N ^2^ (CC)	SD ^4^	*p*-Value
*Body weight*						
31	711 g	283	720 g	239	144	NS ^5^
52	1641 g	228	1627 g	219	284	NS
77	2878 g	187	2707 g	197	325	***
*Daily weight gain*						
31–52	43.2 g/d	228	42.9 g/d	218	11.1	NS
53–77	47.7 g/d	182	42.2 g/d	197	8.3	***
31–77	46.3 g/d	187	43.1 g/d	196	6.0	***
*Mortality*						
31–52	18%	280	8%	240	11.7	**
53–77	11%	275	9%	240	4.5	NS
31–77	29%	275	17%	240	12.4	***
*Morbidity*						
31–77	26%	283	23%	240	7.2	NS
*Rhinitis*						
31–77	16%	283	10%	240	7.9	NS
*Conjunctivitis*						
31–77	3%	283	3%	240	3.3	NS
*Diarrhea*						
31–77	10%	283	11%	240	5.0	NS

^1^ NC = New housing conditions; ^2^ N = No. of rabbits; ^3^ CC = Conventional housing conditions; ^4^ SD = standard deviation, ^5^ NS = not significant; ** = *p* ≤ 0.01, ***= *p* ≤ 0.001.
